# Effects of cytokine signaling inhibition on inflammation-driven tissue remodeling

**DOI:** 10.1016/j.crphar.2021.100023

**Published:** 2021-04-10

**Authors:** Rebecca Bignold, Jill R. Johnson

**Affiliations:** a School of Health and Life Sciences, Aston University, Birmingham, UK; b College of Health and Life Sciences, Aston University, Birmingham, UK

**Keywords:** Fibrosis, Cytokine, Inhibition, IL-1, TNF-α, IL-6, TGF-β

## Abstract

Fibrosis is a common condition that can affect all body tissues, driven by unresolved tissue inflammation and resulting in tissue dysfunction and organ failure that could ultimately lead to death. A myriad of factors are thought to contribute to fibrosis and, although it is relatively common, treatments focusing on reversing fibrosis are few and far between. The process of fibrosis involves a variety of cell types, including epithelial, endothelial, and mesenchymal cells, as well as immune cells, which have been shown to produce pro-fibrotic cytokines. Advances in our understanding of the molecular mechanisms of inflammation-driven tissue fibrosis and scar formation have led to the development of targeted therapeutics aiming to prevent, delay, or even reverse tissue fibrosis. In this review, we describe promising targets and agents in development, with a specific focus on cytokines that have been well-described to play a role in fibrosis: IL-1, TNF-α, IL-6, and TGF-β. An array of small molecule inhibitors, natural compounds, and biologics have been assessed in vivo, in vivo, and in the clinic, demonstrating the capacity to either directly interfere with pro-fibrotic pathways or to block intracellular enzymes that control fibrosis-related signaling pathways. Targeting pro-fibrotic cytokines, potentially via a multi-pronged approach, holds promise for the treatment of inflammation-driven fibrotic diseases in numerous organs. Despite the complexity of the interplay of cytokines in fibrotic tissues, the breadth of the currently ongoing research targeting cytokines suggests that these may hold the key to mitigating tissue fibrosis and reducing organ damage in the future.

## Graphical abstract

**Figure:**
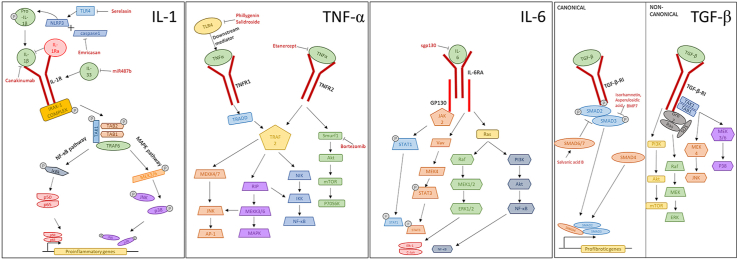

## 1 Background

Fibrosis is a widespread condition that can affect all areas of the body. It involves the scarring of tissue, which is often accompanied by increased tissue inflammation, and results in defective tissue and organ failure that could lead to death. A myriad of factors are thought to contribute to fibrosis and, although it is relatively common, treatments focusing on reversing fibrosis are few and far between. Fibrosis involves a variety of cells, including immune cells such as monocytes/macrophages and leukocytes, which are thought to produce pro-fibrotic cytokines (Wynn and Vannella, 2016). In normal wound healing, these cells coordinate the removal of cellular debris and attract other cell types such as endothelial cells, epithelial cells and fibroblasts to initiate the wound healing process. Endothelial cells contribute to revascularizing the new tissue, fibroblasts differentiate into myofibroblasts and deposit structural proteins such as collagen, and epithelial cells regenerate the tissue. However, in fibrosis, dysregulated myofibroblasts can cause excessive collagen deposition, resulting in the formation of scar tissue and leading to fibrotic organs (Wynn, 2008). These myofibroblasts can originate from existing fibroblasts, but also from epithelial or endothelial to mesenchymal transition as well as through the differentiation of tissue resident mesenchymal progenitor cells, i.e. pericytes (Micallef et al., 2012; Rowley and Johnson, 2014; Kalluri and Weinberg, 2009).

In fibrosis, a number of signaling cascades are modified by changes in the microenvironment, triggered by cytokines. Initially, this involves pro-inflammatory cytokines such as IL-1, IL-6, TNF-α and TGF-β being released from damaged epithelial cells (Li et al., 1998). This stimulates an immune response that results in additional cytokines being released from macrophages (Wynn and Vannella, 2016). The release of these cytokines results in the activation or deactivation of pathways, including the PI3K/Akt pathway, NFκB pathway and SMAD/BMP signaling. This increases the formation of scar tissue and tissue remodeling caused by resident myofibroblasts (Hinz et al., 2012).

**Fig. 1:**
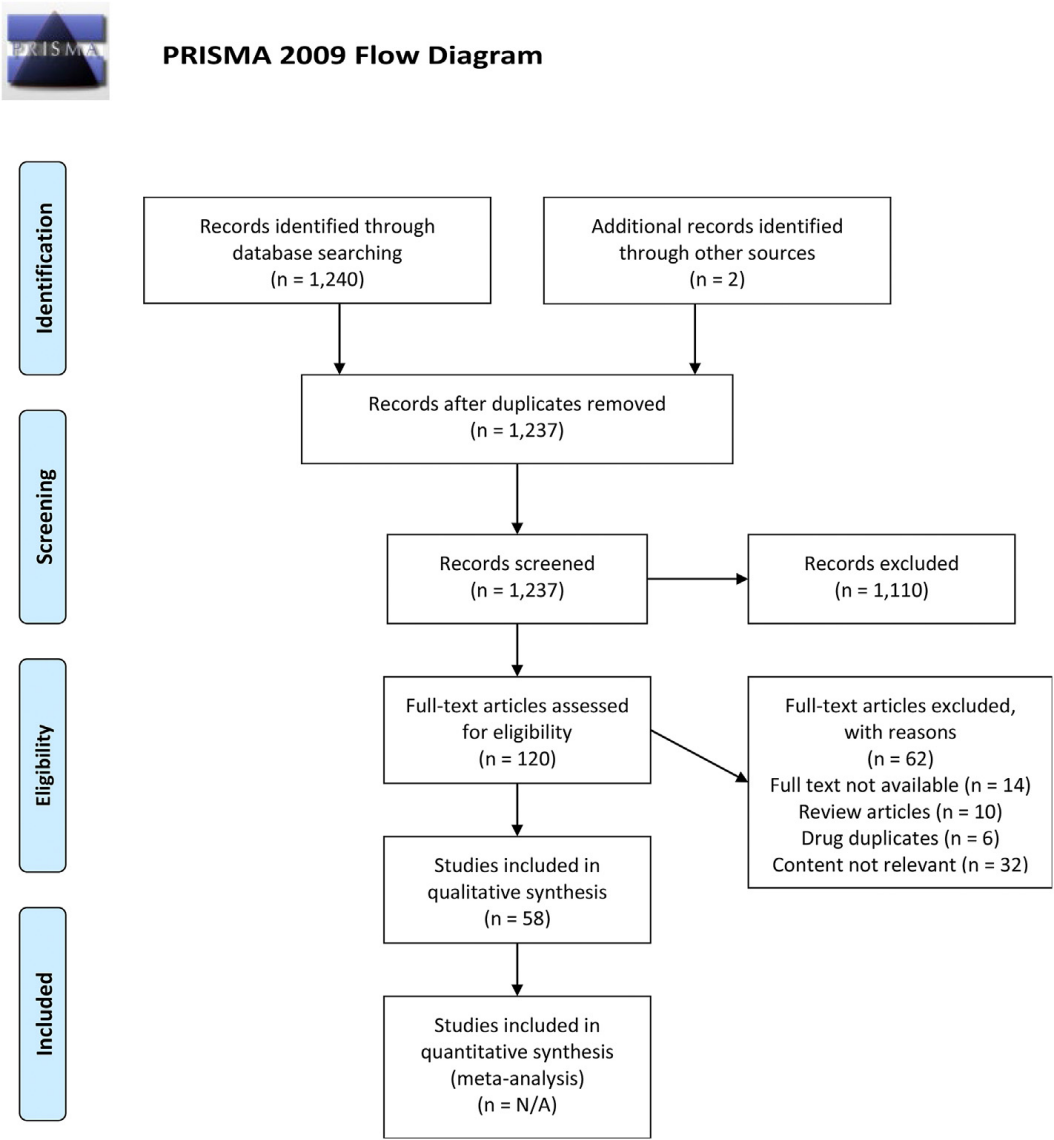
PRISMA flow diagram delineating the search strategy used in the preparation of this review.

As any tissue or organ could be subject to fibrosis; therefore, establishing an effective treatment to prevent or resolve is essential for improving organ function and overall quality of life in patients. For this reason, multiple drugs and inhibitors are currently in development for treating fibrosis in specific organs. However, due to the complexity of the pathways involved and the various functions of these organ, many treatments are not effective in all cases of fibrosis. In an effort to address the inflammatory etiology of organ fibrosis, a number of investigations have been performed to assess the utility of cytokine inhibition in mitigating organ fibrosis. These efforts have mainly focused on four cytokine families: IL-1, TNF-α, IL-6 and TGF-β. This review will describe the roles of these cytokines in the development of fibrosis and highlight utility of cytokine inhibition in reversing tissue scarring (Fig. 1).

## 2 The IL-1 family

**Fig. 2:**
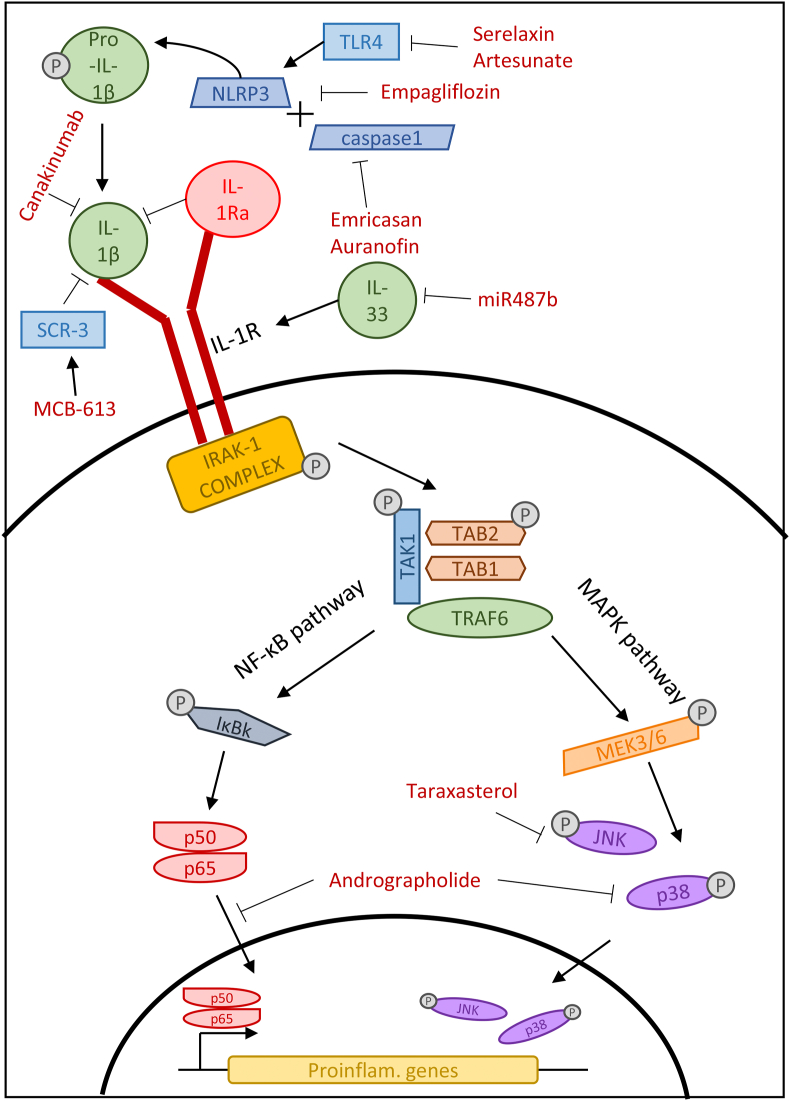
IL-1 family signaling pathways in fibrosis and the mechanism of action of IL-1 pathway inhibitors.

Interleukins are signaling molecules produced by leukocytes and are crucial mediators of the immune response. Many members of the IL-1 family are regarded as pro-inflammatory interleukins and therefore increase the immune response to stimuli. This increase could drive inflammation and exacerbate fibrosis. Reversing this effect using IL-1 inhibitors may reduce fibrosis in the effected organ (Fig. 2).

IL-1β is the most abundant cytokine in the IL-1 family and circulates as the precursor pro-IL-1β. The pro-IL-1β protein is matured by the NLRP3 inflammasome and the activation of caspase-1 (He et al., 2016). Cáceres et al. (2019) inhibited the NLRP3 inflammasome using the drug serelaxin, which is a recombinant form of the protein relaxin. They demonstrated that serelaxin treatment of cardiac myofibroblasts inhibited TLR4, which resulted in the NLRP3 inflammasome being unable to form and therefore IL-1β remaining in its inactive form. They also suggested that this prevented myofibroblast differentiation and reduced collagen deposition. This study implied that in vivo treatment with serelaxin may reduce remodeling and scar tissue formation in cardiac fibrosis (Cáceres et al., 2019). Serelaxin has also been used to treat fibrosis in an OVA-driven model of allergic airway disease. It was able to reduce fibrosis and facilitate bronchodilation, likely through its ability to encourage collagen turnover through an increase in MMPs, which along with the degradation of ECM proteins, is able to reduce the effect of other pro-fibrotic cytokines (Lam et al., 2016, 2018; Royce et al., 2009).

As well as seralaxin, studies have suggested that the drug Empagliflozin also targets the formation of the NLRP3 inflammasome. Sukhanov et al. identified the role of another cytokine (IL-17) on inflammasome formation and subsequent IL-1β activation (Sukhanov et al., 2021). They showed that, through the inhibition of IL-17, various downstream effects can be attenuated, such as the migration and proliferation of aortic smooth muscle cells in vascular proliferative disease. The association between IL-17 and IL-1β activation was confirmed through an increase of IL-1β mRNA and secreted protein, which subsequently induced cell migration and proliferation. They also highlighted that the relationship between IL-17 and the NLRP3 inflammasome may be dependent on NF-κB and AP-1, which are mediated by TRAF3IP2. Empagliflozin treatment was shown to prevent the increase of IL-1β and NLRP3 expression and reduce the proliferation and migration that was induced by IL-17 (Sukhanov et al., 2021). Targeting the proliferation and migration that is seen in vascular proliferative disease as well as many other fibrotic conditions by drugs such as empagliflozin could reduce the damaging effect mesenchymal cells have in inflamed tissue.

A natural inhibitor of IL-1β also exists, known as IL-1Ra, which may be utilized in its recombinant form (anakinra) to reverse fibrosis. It acts as a competitive inhibitor and prevents the activation of the IL-1R1 receptor, therefore preventing the activation of NF-κβ via IL-1β. A recent in vivo study was performed on mice with alcohol-induced liver disease showing that treatment with recombinant IL-1Ra reduced organ fibrosis, assessed by evaluating the expression of PIIINP, a form of procollagen that is often used as a marker for fibrosis (Lotfy et al., 2019). These results corroborated an earlier study showing that anakinra reduced levels of IL-1β in mice with alcoholic liver disease (Petrasek et al., 2012), indicating that this inhibitor may be a potential treatment for alcohol-induced liver fibrosis.

Another study testing an inhibitor targeting the IL-1β pathway in liver fibrosis was performed on mice with non-alcoholic steatohepatitis. Barreyro et al. (2015) used the pan-caspase inhibitor emricasan to target caspase-1, one of the essential proteins needed for IL-1 maturation. By assessing markers of liver damage, i.e. aspartate aminotransferase and alanine aminotransferase, these investigators suggested that emricasan can reverse liver fibrosis induced by a high-fat diet. Inflammatory markers were also tested for, including IL-1β, with emricasan-treated mice expressing significantly less IL-1β when measured by qPCR (Barreyro et al., 2015). This suggests that it is possible to inhibit IL-1β by preventing its maturation and forcing it to remain in the inactive pro-IL-1β form.

The arthritis drug auranofin has also been investigated in the treatment of non-alcoholic fatty liver disease (Hwangbo et al., 2020). Through staining of liver tissue in a mouse model of fatty liver disease, it was shown that auranofin treatment reduced the number of circulating macrophages, which would decrease the amount of inflammatory cytokines secreted. It was confirmed via Western blot that auranofin reduced the secretion of caspase-1 and subsequently IL-1β compared to the control. This was also shown to be due to the reduction of NLRP3 inflammasome following treatment. Auranofin likely interrupts the formation of the NLRP3 inflammasome in fibrotic liver tissue resulting in the activation of IL-1β by caspase-1 (Hwangbo et al., 2020). The upstream target of auranofin encourages a widespread effect and increased the effectiveness of the drug.

An existing drug, artesunate, normally used to treat malaria has been shown to combat fibrotic diseases, specifically hepatic ischemia (El-Sayed Ghoneim et al., 2020). It has been shown that treatment of artesunate in rats with hepatic ischemia reduced the expression of the NLRP3 protein complex as well as caspase-1, the protein responsible for the activation of IL-1β. This was confirmed via Western blot as well as the subsequent reduction of circulating IL-1β. This downstream inhibition was likely to be determined by the disruption of TLR4, TRAF6 and NF-κB signaling shown via ELISA assay. It was also shown that artesunate has an overall protective effect against hepatic ischemia as liver injury scores determined through histopathological staining were significantly lowered (El-Sayed Ghoneim et al., 2020). The repurposing of existing drugs to treat fibrosis is a lucrative pathway in drug development as thorough safety analyses have already been performed.

Studies have also focused on IL-1β in renal fibrosis. Masola et al. (2019) performed an in vitro study using human renal proximal tubular epithelial cells (HK-2) and hepatic stellate cells and (LX-2, i.e. pericytes) to test the impact of IL-1β inhibition on fibrosis. This study focused on epithelial-to-mesenchymal transition (EMT) as a driver of fibrosis and a source of myofibroblasts, which is induced by the release of inflammatory cytokines and growth factors and leads to the overproduction of collagen and tissue remodeling (Fragiadaki and Mason, 2011). It was observed that treatment with IL-1β induced EMT, demonstrated by increased expression of the fibrotic markers fibronectin, vimentin and α-smooth muscle actin (α-SMA). Crucially, treatment with the IL-1β inhibitor canakinumab reversed the expression of these markers as well as that of a critical mediator of EMT, MMP-2. Moreover, canakinumab also attenuated the induction of a myofibroblast phenotype caused by TGF-β treatment, suggesting that the inhibition is not specific to IL-1β, indicating that this therapeutic modality may also be promising for resolving kidney fibrosis (Masola et al., 2019).

Renal ischemia also results in inflammation, oxidative stress and the induction of fibrosis. Taraxasterol, the active compound in the traditional herbal medicine *Taraxacum officinale*, has been investigated for its anti-inflammatory effects (Li et al., 2020). In mice with acute kidney injury, taraxasterol treatment decreased the pathological score of renal tubular injury, indicating a reduction in cell necrosis, tubular dilation and the infiltration of inflammatory cells. This suggests that taraxasterol must interrupt upstream inductors of fibrosis. In this model of kidney injury, macrophage numbers significantly increased along with interstitial fibrosis; these were both attenuated with taraxasterol treatment. along with a reduction in serum IL-1β. This was likely achieved by a reduction in ERK and JNK phosphorylation, thus blocking the downstream effects of IL-1β. It was also suggested that reactive oxygen species are produced following IL-1β signaling, which was abrogated by taraxasterol via the inhibition of MAPK signaling (Li et al., 2020). The combined approach with taraxasterol mitigating both inflammation and oxidative stress seems to be a promising mode of action in reducing tissue remodeling in fibrosis.

Another natural anti-fibrotic drug being explored is andrographolide, a compound isolated from *Andrographis paniculate*; this natural resource has been traditionally used to treat inflammation, although the mechanism of action was unknown. Gupta et al. investigated the effects of this active compound in a mouse model of arthritis manifested as an increase in the infiltration of immune cells in the hind limbs (Gupta et al., 2020). Histopathological analysis suggested that treatment with andrographolide reduced the number of immune cells present and encouraged the regrowth of the synovial lining. Mechanistically, it was found that andrographolide attenuated NF-κB translocation into the nucleus, thereby inducing immune suppression. Along with this, the phosphorylation of p38 and the concentration of serum IL-1β were significantly reduced following andrographolide treatment. This inhibition was shown to decrease paw edema and improve the presentation of arthritic fibrosis (Gupta et al., 2020). By targeting NF-κB, which is involved in many pro-inflammatory and pro-fibrotic signaling cascades, andrographolide may have a beneficial impact in a number of inflammatory and fibrotic diseases.

IL-1β has also been identified as one of the most significant triggers in fibrosis following myocardial infarction. It has been highlighted that IL-1β may contribute to fibroblast differentiation, which would contribute to disordered tissue remodeling. Treating mice with the small molecule MCB-613, a steroid receptor coactivator stimulator,was shown to decrease IL-1β mRNA expression in cardiac fibroblasts following myocardial infarction via single cell analysis (Mullany et al., 2020). This was also shown in an inflamed microenvironment induced by M1 macrophages. Mullany et al. suggested that MCB-613 works via the upregulation of SRC-3, which is an inhibitor of IL-1β activity in macrophages. This, in turn, would reduce the concentration of IL-1β in the microenvironment, thus reducing the differentiation of cardiac fibroblasts. This was mechanistically confirmed with in vitro experiments. Treatment with MCB-613 was shown to greatly reduce fibrotic tissue in murine hearts following myocardial infarction, illustrating the overall effect of IL-1β inhibition and the reduction of inflammation in the microenvironment (Mullany et al., 2020). This study highlights the contribution of fibroblasts in cardiac remodeling as well as the potential of small molecule stimulators in the reduction of fibrotic tissue.

IL-33 is another member of the IL-1 family of pro-inflammatory interleukins. It has long been thought to contribute to fibrosis, in particular in allergic inflammation. This is due to its ability to activate immune cells such as macrophages, eosinophils, cytotoxic T cells, and group 2 innate lymphoid cells upon cell damage (Chan et al., 2019; Kondo et al., 2008; Bonilla et al., 2012). These activated immune cells promote the fibrotic microenvironment and encourages the subsequent production of further pro-fibrotic cytokines. The inhibition of IL-33 would theoretically prevent the downstream activation of pro-fibrotic cytokines thus reducing fibrosis. One study has investigated the role of IL-33 expression in rats with chronic heart failure (Wang et al., 2017). The IL-33/ST2 signaling axis was inhibited using the microRNA miR487b, which was found to reduce the expression of the IL-33 protein, resulting in improved cardiac morphology and reduced collagen expression in an in vivo model of chronic heart failure induces by coronary artery occlusion. The use of microRNAs to inhibit cytokines and the downstream signaling pathways may therefore be a fruitful avenue to pursue in ongoing efforts to attenuate tissue fibrosis.

## 3 TNF-α

**Fig. 3:**
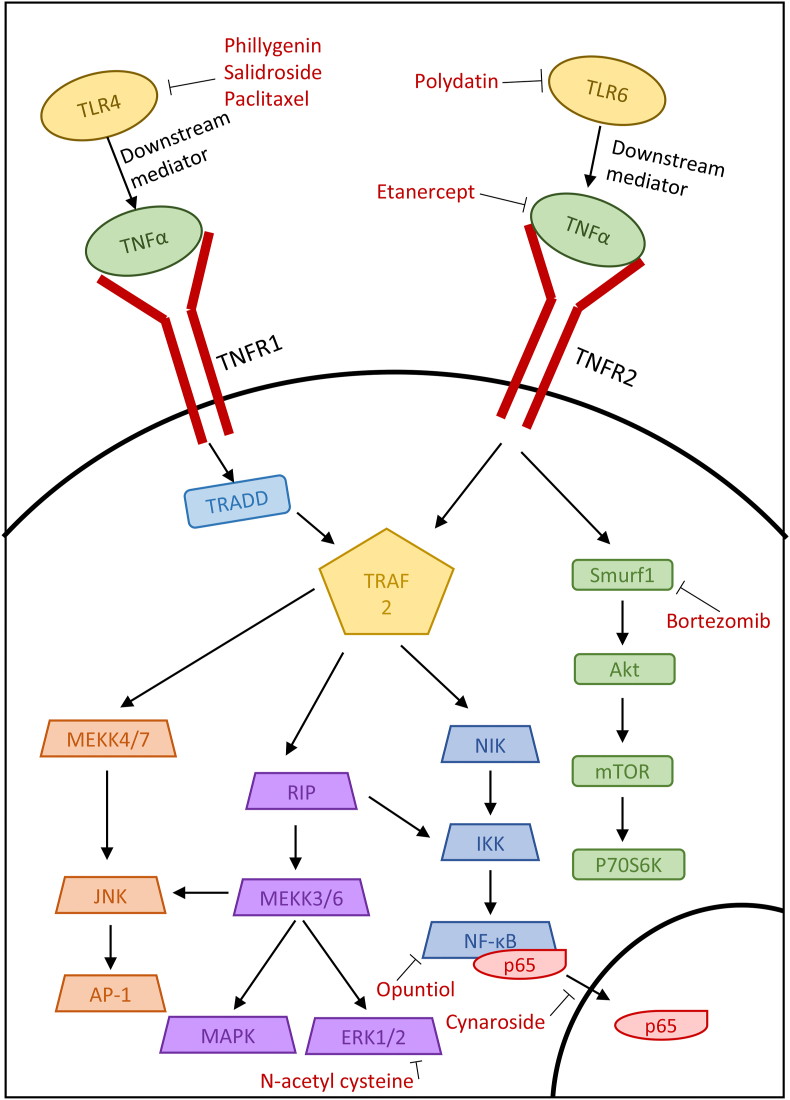
TNF-α signaling pathways in fibrosis and the mechanism of action of TNF-α pathway inhibitors.

TNF-α is produced during inflammation by immune cells such as macrophages and monocytes. TNF-α has various roles within the body, including pathogen resistance, anti-tumor immunity and sleep regulation. It affects these processes by activating a variety of pathways, including NF-κB, Erk, MAPK and PLA_2_ (Idriss and Naismith, 2000). The important roles of TNF-α in regulating inflammation explains why it is also involved in fibrosis as the two often come hand in hand (Fig. 3).

Following organ transplantation, extensive fibrosis often occurs, i.e. chronic transplant rejection. Zhou et al. (2019) investigated the effect of a TNF-α inhibitor to prevent EMT, a key driver of interstitial renal fibrosis. They showed that treatment of HK-2 ​cells with TNF-α caused an increase in the EMT markers α-SMA and fibronectin via the activation of Smurf1, an e3 ubiquitin ligase that has been found to be involved in cell migration and polarity as well as EMT through its interaction with the TGF-β canonical pathway and NF-κβ signaling (Zhou et al., 2019). The anti-cancer drug bortezomib, a proteasome inhibitor, has also been shown to target Smurf1, a HECT-type E3 ubiquitin ligase that plays a critical role in vertebrate development (Koefoed et al., 2018), thereby reducing the phosphorylation of Akt and inhibiting EMT. Moreover, bortezomib was found to reduce inflammation and fibrosis after orthotopic kidney transplantation in rats (Zhou et al., 2019). This suggests that existing cancer drugs may be useful tools in mitigating fibrosis (Wishart et al., 2006).

As previously mentioned, TNF-α can activate the MAPK pathway via NF-κB, a critical pathway mediating inflammation. In addition, TNF-α is thought to interact with TLR4 and modulate its expression, with variations in TLR4 signaling being an important factor in many diseases (Simonaro et al., 2010). Using the unilateral ureteric obstruction (UUO) mouse model of renal interstitial fibrosis, Li et al. (2019) tested the natural drug salidroside and its ability to inhibit the NF-κB pathway. They showed that salidroside reduced the inflammatory markers TNF-α, IL-6 and IL-1β in vivo. It also reduced the levels of both collagen I and III in the ECM and reduced EMT via the downregulation of TGF-β, α-SMA and vimentin. They postulated that salidroside had these effects by inhibiting TLR4, which subsequently reduced the expression of the downstream molecules NF-κB and p-ERK, shown via Western blot (Li et al., 2019).

Naturally sourced compounds have also been suggested to affect the TNF-α/NFκB pathway. The drug phillygenin is obtained from the plant *Forsythia suspensa* and is often used in traditional Chinese medicine. In an in vitro study investigating the induction of a fibrotic phenotype in LX-2 hepatic stellate cells, phillygenin reduced the expression of IL-1β, IL-6 and TNF-α, suggesting that it has anti-inflammatory properties (Hu et al., 2020). Western blot experiments also indicated that treatment with phillygenin mitigated the induction of fibrosis markers, as it reduced the expression of α-SMA and collagen 1 (Hu et al., 2020). It was also found in this study that phillygenin binds directly with TLR4 in order to inhibit the downstream signaling cascade (Hu et al., 2020). This study highlights the possibility of using natural drugs to reverse tissue fibrosis and inflammation.

Periodontal fibrosis has been treated using the flavonoid cynaroside and the mechanisms behind its activity have been explored (Lee et al., 2020). The effects of cymaroside were tested in vitro on human periodontal ligament cells. LPS was used as an inductor of fibrosis and increased the expression of TNF-α, iNOS and COX-2. These increases were reversed with cymaroside, observed via Western blot, and were also observed in RAW264.7 ​cells. It was hypothesized that this effect was mediated through the inhibition of NF-κB, as cynaroside prevented the translocation of the NF-κB p65 subunit to the nucleus. This prevent the resulting signalling cascade and impaired the induction of fibrosis. It was also shown that treatment with cynaroside encouraged the differentiation of periodontal ligament cells into a more osteogenic morphology, so this flavonoid may be useful for repairing damaged teeth (Lee et al., 2020). By preventing the action of TNF-α through the arrest of NF-κB as well as reforming bone tissue, cynaroside is a promising treatment for periodontal fibrosis.

Chronic respiratory disease often involves severe inflammation and, in chickens, can be caused by mycoplasma infection. Zou et al. (2020) proposed using polydatin, a resveratrol glycoside isolated from *Polygonum cuspidatum*, to treat this inflammation. Damaged lungs showed increased numbers of inflammatory cells, shedding of epithelial cells and alveolar congestion. These changes were not present in lungs treated with polydatin, suggesting a molecular intervention with this anti-fibrotic compound. Polydatin was also shown to reduce TLR6 expression in a dose-dependent manner. TLR6 activates the NF-κB pathway, with Zou showing that polydatin treatment resulted in a decrease in nuclear NF-κB translocation. Proinflammatory cytokines, including TNF-α, were also markedly decreased following polydatin treatment, suggesting that polydatin successfully blocks the effect of proinflammatory cytokines (Zou et al., 2020). Upstream targeting by polydatin allows the inhibition of a variety of cytokines and thus further reduces the impact of inflammation on tissue morphology.

Opuntiol, the active ingredient from *Opuntia ficus-indica*, is known for its anti-UV properties and is being investigated as a treatment against UVA-induced skin fibrosis (Kandan et al., 2020). Overall, opuntiol reduced the clinical score of fibrosis severity in mice following UVA exposure. In addition, pretreatment with opuntiol prevented epidermal hyperplasia and loss of collagen. This suggests that opuntiol has a molecular protective effect which results in the prevention of UV damage. Using Western blot, the location of NF-κB and AP-1 was determined, demonstrating that opuntiol prevented the nuclear translocation of NF-κB and AP-1, thereby preventing transcription. With immunohistochemistry, it was highlighted that topical treatment with opuntiol prevented the build-up of inflammatory proteins such as TNF-α, COX-2 and iNOS. This suggests that the blockade of the NF-κB prevented TNF-α-mediated inflammation from further exacerbating fibrosis (Kandan et al., 2020). The effect of UV on pro-inflammatory cytokines was effectively prevented by opuntiol, suggesting that it may be a good topical treatment for UV-induced fibrosis.

Son et al. highlighted the positive effects of paclitaxel on diabetic nephropathy via targeting TNF-α and TLR4 (Son et al., 2020). An in vitro study was carried out using podocytes to simulate diabetic nephropathy. Podocyte injury induced by palmitate exposure increased the mRNA levels of TNF-α and TLR4, but this increase was abrogated by treatment with paclitaxel. Along with this, reactive oxygen species and NOX4, which controls reactive oxygen species production, were increased after injury and reduced following treatment. This intervention may have contribute to a reduction in podocyte damage, as paclitaxel treatment prevented F-actin rearrangement, reduced VEGF mRNA levels and increased nephrin expression (Son et al., 2020).

N-acetyl cysteine is thought to inhibit the formation of ROS, but has been suggested to also have an anti-fibrotic effect. In a study by Honma et al., renal interstitial fibrosis was simulated in mice in order to investigate the effect of N-acetyl cysteine on renal fibrosis (Honma et al., 2020). The overexpression of TNF-α observed in the fibrotic kidney was attenuated following N-acetyl cysteine treatment, along with a reduction in the mRNA levels of fibrosis-associated collagen III. The MAPK pathway was also affected by N-acetyl cysteine, as this intervention reduced ERK1/2 phosphorylation, although the JNK and p38 pathways were not affected. Honma et al. concluded that the intervention with N-acetyl cysteine contributed to a reduction in TNF-α and subsequent reduction in collagen production (Honma et al., 2020). As ERK1/2 is a member of several cytokine pathways, this downstream intervention may be key to effectively combating fibrosis.

Clinical trials have been undertaken using the TNF-α inhibitor etanercept in order to treat cytotoxic T lymphocyte-mediated severe cutaneous adverse reactions (SCARs) (Wang et al., 2018). It was hypothesized that TNF-α was responsible for the heightened immune response in SCARs as well as the death of keratinocytes. As etanercept is a TNF-α antagonist, it was able to reduce the levels of TNF-α as well as the cell death mediator granulysin both in vitro and in vivo. In human trials, Wang et al. also demonstrated that etanercept treatment caused skin lesions to heal faster and with less inflammation than with standard corticosteroid treatment. They also showed that etanercept treatment increased the population of regulatory T cells in the lesion, which also reduced patient mortality (Wang et al., 2018). The ability of TNF-α to modulate the immune system under inflammatory and fibrotic conditions highlights the possibility of targeting TNF-α for anti-inflammatory and anti-fibrotic therapies.

## 4 IL-6

**Fig. 4:**
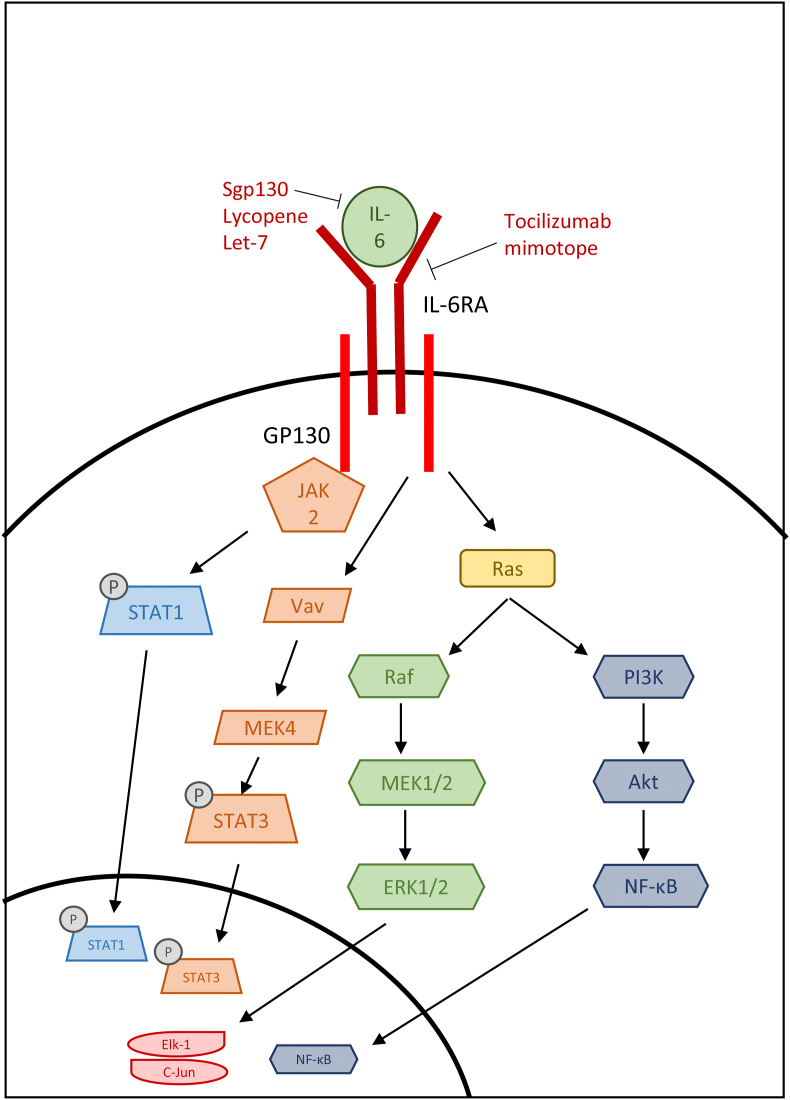
IL-6 signaling pathways in fibrosis and the mechanism of action of IL-6 pathway inhibitors.

One of the downstream mediators of the aforementioned IL-1 family is the inflammatory interleukin IL-6 (Fig. 4), which plays an active role in the modulation and activation of the immune response; defects in IL-6 signaling play a role in many inflammatory and autoimmune diseases (Hirano, 2010). IL-6 activates the JAK/STAT3 pathway and the SHP2/Gab/MAPK pathway and interacts with the NLRP3 inflammasome (Hirano, 2010; Del Campo et al., 2018). Its ability to induce monocyte differentiation into macrophages and activate Th2 and Th17 immune cells also indicates its influence on inflammation and fibrosis (Su et al., 2017; Fielding et al., 2014). The activation of Th2 and Th17 can be explored using different models of fibrosis, with more mild fibrosis being Th2-driven, whilst severe fibrosis is characterized by Th17 responses, more often seen in unresolved, chronic infections and inflammatory diseases (Zhang and Zhang, 2020). For example, the house dust mite model of allergic airway disease induces a mainly Th2 immune response, but extended aeroallergen exposure leads to a more Th17-skewed response with extensive tissue remodeling characterized by EMT (Johnson et al., 2004, 2011, 2015).

With the signaling pathways of IL-6 being so varied, there have been several different methods of intervention when therapeutically targeting IL-6. A study by Chou et al. (2018) investigated the effects of IL-6 in aldosterone-induced cardiac fibrosis. They used a natural existing inhibitor of IL-6, sgp130, which can block IL-6 and its soluble form from binding and inducing the trans-signaling pathway in endothelial cells. By analyzing the expression of fibronectin and collagen-1, they suggested that this inhibitor mitigates fibrosis promoted by IL-6. In vivo, they also showed that recombinant gp130 decreased cardiac fibrosis by observing reduced ventricular fibrosis following aldosterone infusion in mice (Chou et al., 2018). This study highlighted the impact of IL-6 on fibrosis and the potential for therapeutics that interrupt the IL-6 trans-signaling pathway.

Fatty liver disease is another fibrotic condition that is exacerbated by IL-6. In rats with fatty liver disease, serum levels of IL-6 increased significantly, but were decreased following lycopene treatment (Saeeda et al., 2020). Similarly, NF-κB levels were increased in diseased tissue and decreased with lycopene. Lycopene treatment also prevented structural changes such as increased α-SMA expression. Further staining also concluded that lycopene reduced the number in inflammatory cells present in the effected tissue and mitigated collagen deposition. Saeed et al. concluded that IL-6 levels are an indicator of insulin resistance and liver damage, and that a reduction in circulating IL-6 leads to a reduction in NF-κB activation and therefore a reduction in structural remodeling (Saeeda et al., 2020). With lycopene also targeting insulin resistance as well as pro-inflammatory cytokine signaling, this two-pronged approach may be an effective treatment for fatty liver diseases and associated fibrosis.

Renal fibrosis is also affected by IL-6 signaling and studies have identified a tocilizumab mimotope that can target the IL-6 receptor in order to suppress this signaling pathway (Yang et al., 2020). Via a vaccine strategy, the mimotope was administered to mice with renal fibrosis established using the UUO model in order induce inhibitory antibodies that target IL-6R. Mimotope treatment decreased levels of fibronectin, collagen and α-SMA compared to untreated diseased tissue via the modulation of IL-6 signaling. This treatment also suppressed the differentiation of macrophages in the kidneys, further reducing the secretion of pro-inflammatory cytokines. Vaccination also had downstream effects, as it reduced the phosphorylation of ERK, thereby preventing that pathway from contributing to fibrosis. Further injury to the kidney could also be at least partly prevented by the tocilizumab mimotope, as it slightly reduced the levels of ferroptosis, which leads to additional kidney injury. Although the mimotopes successfully inhibited pERK, no change was observed in the levels of phosphorylated STAT3, so only part of the IL-6 pathway was successfully inhibited (Yang et al., 2020). Despite this, the effects of the tocilizumab mimotope showed promising effects in terms of preventing kidney fibrosis.

## 5 TGF-β

**Fig. 5:**
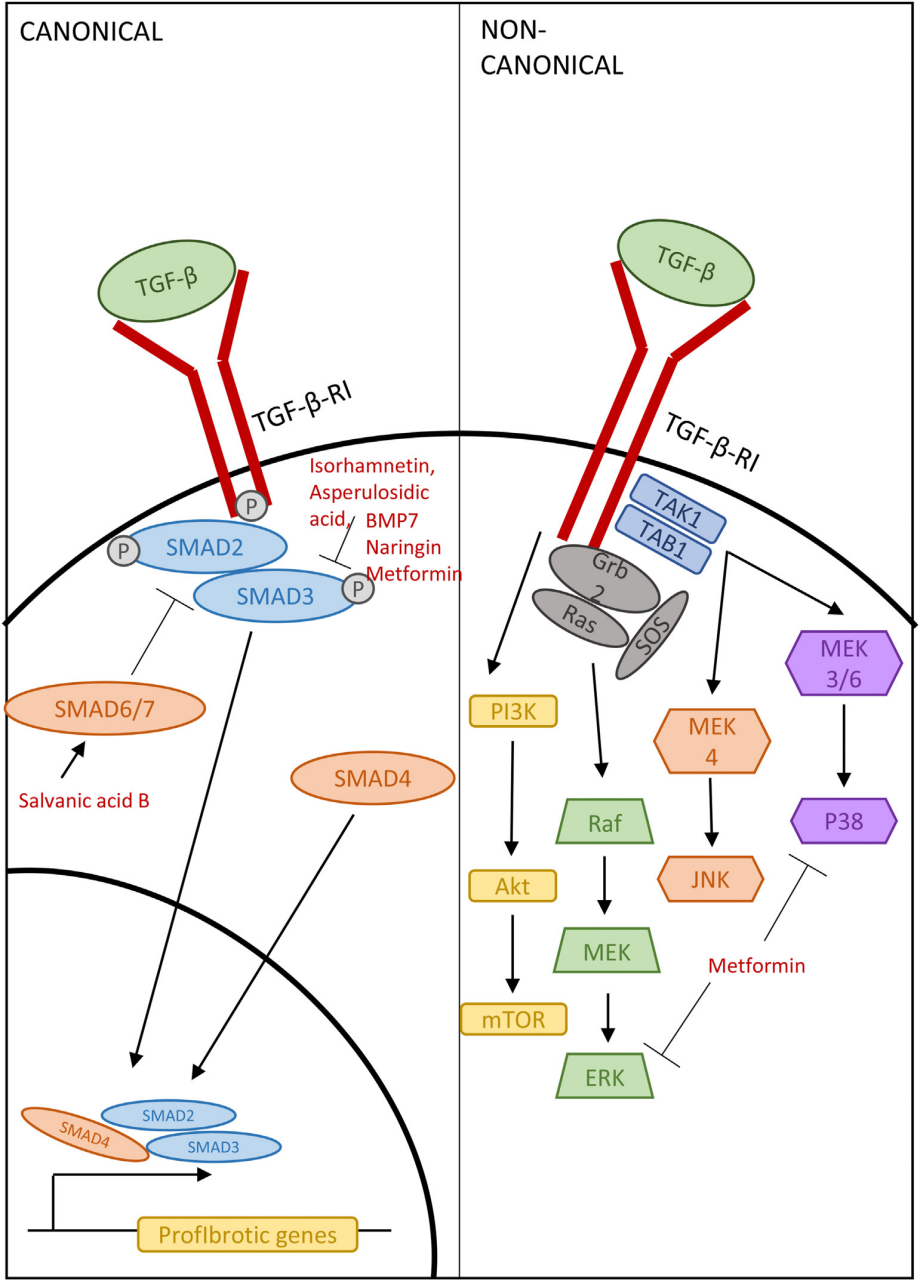
TGF-β signaling pathways in fibrosis and the mechanism of action of TGF-β pathway inhibitors.

It is widely recognized that TGF-β is one of the main cytokines involved in fibrosis (Fig. 5). It is upregulated in most fibrotic diseases; however, paradoxically, it also has anti-inflammatory effects. This is due to two major signaling pathways, i.e. the canonical pathway mediated by a cascade of Smad protein interactions and the non-canonical pathway with activation of mTOR, Erk, JNK and p38 signaling. However, due to its widespread roles in proliferation, differentiation and various other homeostatic functions, inhibiting TGF-β upstream may induce many side effects. Therefore, any drugs targeting TGF-β are likely to instead target the Smad signaling pathway (Kubiczkova et al., 2012; Biernacka et al., 2011; Meng et al., 2016).

A study by Yang et al. isolated the natural TGF-β inhibitor isorhamnetin from the leaves of the plant *Oenanthe javanica* (Yang et al., 2016). They showed that treatment with 50–100 ​μM isorhamnetin reduced the expression of fibrotic markers α-SMA and PAI-1 in TGF-β-treated hepatic stellate cells. Isorhamnetin also decreased Smad binding element activity and greatly inhibited the phosphorylation of Smad2 and Smad3, suggesting that it targets this stage of the canonical TGF-β signaling cascade. They also highlighted the protective effect of isorhamnetin against oxidative stress by preventing TGF-β-induced reactive oxygen species production and by activating the antioxidant protein Nrf2 (Yang et al., 2016). This two-pronged approach is promising as it combats two triggers of fibrosis whilst not blocking TGF-β activity completely, which should reduce possible side effects.

Another study on the role of TGF-β in liver fibrosis utilized a mouse model to investigate the relationship between TGF-β and another member of the TGF family, BMP7 (Zou et al., 2019). These authors observed that mice with induced liver fibrosis had upregulated levels of TGF-β and Smad3, and downregulated BMP7 and Smad1/5/8. This suggests that Smad1/5/8 signaling might inhibit the fibrotic effects of TGF-β via BMP7 (Guo et al., 2017). They also showed that, in vitro, a high dose BMP7 delivered to TGF-β-treated hepatic stellate cells reduced the production of the myofibroblast markers α-SMA and collagen I and moreover inhibited p38-induced migration and proliferation. This suggests that BMP7, via Smad1/5/8, inhibits hepatic stellate cell activation and mitigates fibrosis through a reduction in activated myofibroblasts (Zou et al., 2019). The use of exogenous proteins and existing inhibitory signaling cascades highlights further targets for therapeutic intervention without having to introduce molecules that may have off-target effects.

Along with liver fibrosis, kidney fibrosis is another condition which often presents with elevated levels of TGF-β. Lu et al. hypothesized that the naturally occurring compound asperulosidic acid, which has been reported to have anti-fibrotic and anti-inflammatory effects, may interfere with TGF-β signaling in order to ameliorate kidney fibrosis (Lu et al., 2019). These authors found that, in rats with unilateral ureteral obstruction, asperulosidic acid reduced markers of renal damage, blood urea nitrogen, uric acid and urinary protein, suggesting that the treatment reduced kidney fibrosis overall. More specifically, they showed that asperulosidic acid decreased the expression of the inflammatory cytokines TNF-α, IL-1β and IL-6, suggesting that asperulosidic acid can modulate inflammation through the inhibition of NF-κB. Asperilosidic acid also reduced the expression of TGF-β and α-SMA, likely due to the regulation of pro-fibrotic Smad2/3/4 signaling (Lu et al., 2019). By inhibiting both the inflammatory pathway and TGF-β-induced pathway, the overall inhibition of fibrosis should be more effective than treatments that only target one arm of the response. By showing that this treatment has an effect on the overall pathogenesis of kidney fibrosis, it highlights the potential of this treatment and therefore the importance of these targeted processes on the development of fibrosis.

A different acid which is thought to interfere with TGF-β signaling is salvanic acid B. A study performed in a mouse model of myocardial fibrosis confirmed that TGF-β and Smad2/3 were increased during fibrosis, with the antifibrotic Smad7 being decreased (Gao et al., 2019). Following salvanic acid B treatment, this was reversed with decreased TGF-β and Smad2/3 expression and increased Smad7 levels on the mRNA and protein levels. The morphology of cardiac fibroblasts was also investigated, with the spindle shape of TGF-β-treated fibroblasts being modulated by salvanic acid B. Salvanic acid B was also shown to reduce the expression of the extracellular matrix enzymes MMP-2 and MMP-9, indicating that TGF-β induces modulation of the extracellular matrix, a hallmark of fibrosis, and that this can be attenuated by salvanic acid B treatment (Gao et al., 2019). This study suggests that the inhibition of the Smad2/3 protein complex in the TGF-β canonical signaling pathway is key to reducing the effect of TGF-β on the extracellular matrix and perhaps the formation of myofibroblasts. It also touched upon harnessing Smad7, a natural regulator of the TGF-β pathway. By controlling this regulator of TGF-β signaling, it may be possible to dampen its effects in fibrosis.

TGF-β also contributes to interstitial fibrosis in the kidneys. Wang et al. explored the effects of the dihydroflavonoid naringin on TGF-β-induced fibrotic renal epithelial cells (Wang et al., 2021). Cell fibrosis, characterized by the expression of α-SMA, was decreased following treatment with naringin, highlighting its ability to inhibit the fibrotic effect of TGF-β. By targeting the canonical TGF-β pathway and the phosphorylation of Smad2/3, fibrosis was markedly reduced, and downstream mediators of other pro-fibrotic cascades such as NF-κB were also suppressed. To corroborate this, mRNA levels of fibrotic markers TGF-β, α-SMA and collagen 1 were measured in vivo following the induction of renal fibrosis with the UUO model. Treatment with naringin significantly reduced all fibrotic markers both in vivo and in vitro, highlighting its effectiveness as an anti-fibrotic drug. Naringin moreover improved the atrophy of kidney tubular cells following fibrotic injury, which suggests that it may have a clinically useful effect (Wang et al., 2021).

The diabetic drug metformin has been tested in chronic kidney diseases to explore its potential as a TGF-β-targeting anti-fibrotic intervention (Yi et al., 2021). Metformin treatment after adenine-induced renal injury in mice significantly decreased urinary albumin levels, suggesting an improvement in kidney function. It was also shown to decrease fibrosis, as the mRNA levels of both collagen 4 and fibronectin were decreased, highlighting a reduction in extracellular matrix protein accumulation and the prevention of fibrosis. Inflammation was also reduced following metformin treatment, as the number of activated macrophages decreased as well as the levels of macrophage chemotactic protein, which is often used as a marker for renal inflammation. Using Western blot, Yi et al. showed that metformin inhibited the phosphorylation of Smad3 as well as ERK1/2 and p38, thus suppressing both the canonical and non-canonical TGF-β signaling pathways (Yi et al., 2021). By targeting both arms of the TGF-β signaling pathway, metformin may have a considerable effect in terms of mitigating fibrosis.

## 6 Outlook

There are many different types of cytokine inhibitors currently being investigated as potential treatments for fibrosis in various organs. The variety of strategies and targets discussed here indicates the scope of this area of research and the complexity of the systems at play in fibrosis. Some treatments do not directly inhibit the target cytokine and instead target a molecule upstream (in the case of emericasan and serelaxin), thereby preventing the active cytokine from being produced, or downstream mediators (e.g. bortezomib and salidroside) to prevent the signaling cascade and the action of the cytokine from occurring in full. There is also a possibility of harnessing the body’s innate defenses, such as in the case of IL-1Ra and BMP7, and either increasing or modulating their effects in order to combat fibrosis. There is also an increasing number of natural, plant-based compounds being explored for their anti-fibrotic properties, including phillygenin, lycopene and isohamnetin, which have been used in herbalism and traditional medicine systems and have been more recently shown to have scientific promise in the case of treating fibrosis. Despite the complexity of the interplay of cytokines in fibrotic tissues, the breadth of the currently ongoing research targeting cytokines suggests that these may hold the key to mitigating tissue fibrosis and reducing organ damage in the future.

## CRediT authorship contribution statement

**Rebecca Bignold:** performed the systematic review and prepared the manuscript. **Jill R. Johnson:** Conceptualization, Writing – review & editing.

## Declaration of competing interest

The authors declare that they have no known competing financial interests or personal relationships that could have appeared to influence the work reported in this paper.
